# GLP‐1 Receptor Agonists and Facial Aging: Two Overlooked Mechanisms

**DOI:** 10.1111/jocd.71077

**Published:** 2026-07-20

**Authors:** Mingpeng Feng, Xuanli Huang

**Affiliations:** ^1^ Medicine & Technology College Zunyi Medical University Zunyi City Guizhou Province China; ^2^ The Fifth Affiliated (Zhuhai) Hospital of Zunyi Medical University Zhuhai City Guangdong Province China

**Keywords:** adipose‐derived stem cells, collagen degradation, facial aging, GLP‐1 receptor agonists, “Ozempic face”, oxidative stress

Dear Editor,

Glucagon‐like peptide‐1 receptor agonists, including semaglutide, the dual GIP/GLP‐1 receptor agonist tirzepatide, are widely used for type 2 diabetes mellitus (semaglutide 1 or 2 mg weekly; tirzepatide 5, 10, or 15 mg weekly) and, at higher doses or in specific formulations, for chronic weight management (semaglutide 2.4 or 7.2 mg weekly, depending on formulation and regional approval; tirzepatide 5, 10, or 15 mg weekly). These drugs, abbreviated as GLP‐1RAs, have brought an unintended consequence to dermatology clinics, referred to in public discourse as “Ozempic face”, characterized by premature facial hollowing, skin laxity, and an aged appearance [1, 2]. While commonly attributed to rapid fat loss, emerging evidence suggests two additional, independent mechanisms that have received insufficient attention.

The disproportionate nature of facial fat loss provides the first clue. A quantitative study using 3D imaging found that patients receiving semaglutide (2.4 mg weekly) experienced average reductions of 41.8% in temporal fat pads and 69.9% in cheek fat pads. These figures dramatically exceed total body fat reduction, which was approximately 9.2% in the same cohort. The discrepancy suggests that GLP‐1RAs may directly and preferentially target facial adipose depots, rather than merely inducing systemic fat loss. A direct dermal matrix injury cascade further supports this view. A 2025 Mini Review in Endocrine details how GLP‐1RAs act on adipose‐derived stem cells (ADSCs) and fibroblasts, both of which express GLP‐1 receptors [3] (Figure 1A). Receptor stimulation reduces ADSC production of protective cytokines, leading to reactive oxygen species accumulation and oxidative damage to fibroblasts. GLP‐1RAs also reduce glucose uptake by ADSCs, causing ATP depletion and apoptosis. The ADSC stimulation indirectly reduces estrogen production from dermal white adipose tissue, impairing fibroblast stimulation and collagen synthesis. This mechanistic cascade, from adipose‐derived stem cell dysfunction to oxidative stress, subsequent fibroblast injury, and ultimately impaired collagen synthesis, raises the possibility that GLP‐1RAs may promote skin aging via pathways wholly distinct from those mediating volumetric fat loss (Figure 1A). Whether these facial changes are specific to GLP‐1 receptor agonism or extend to the dual GIP/GLP‐1 receptor agonist tirzepatide remains unclear, as comparative data are currently limited.

**FIGURE 1:**
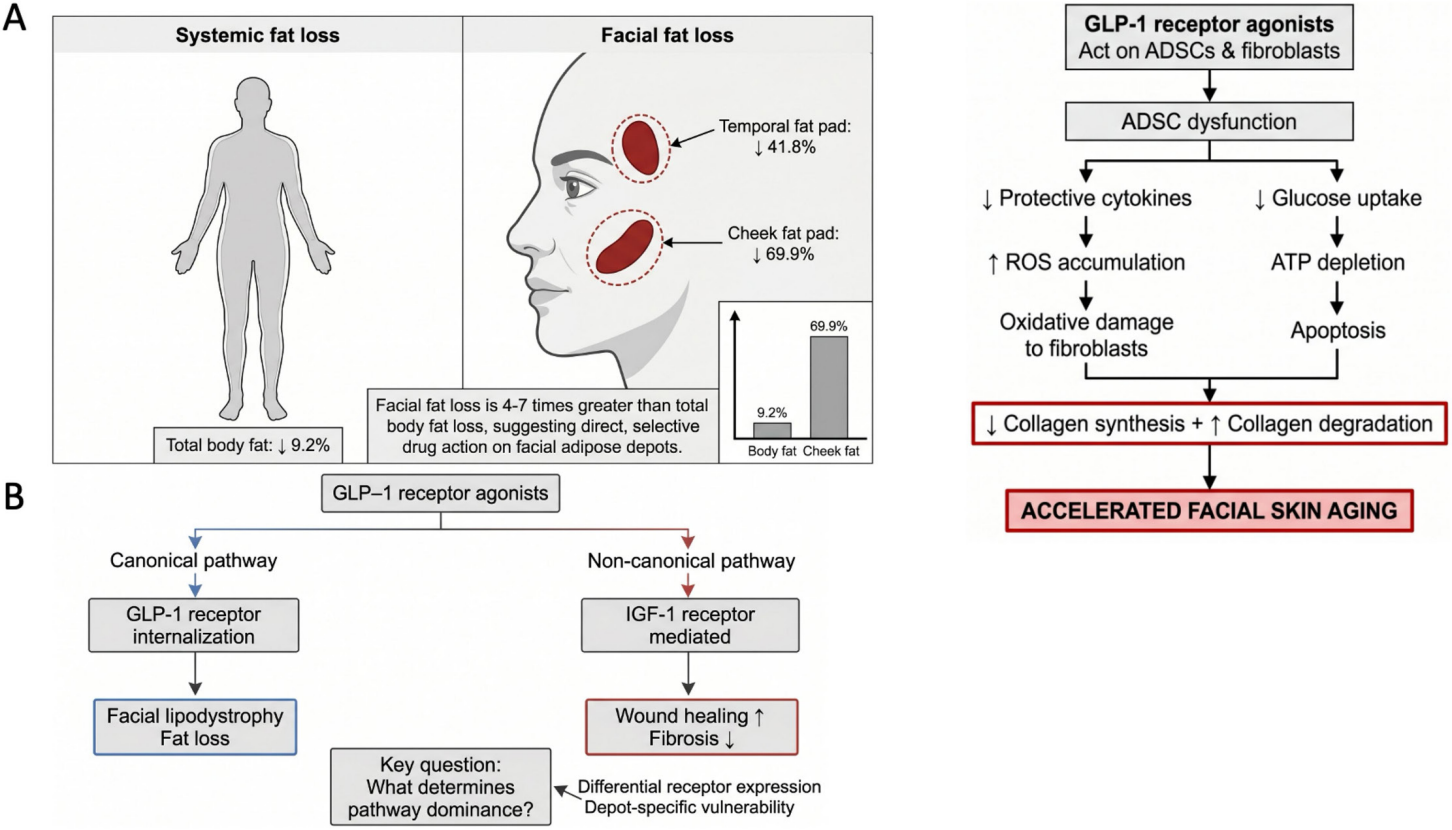
(A) Disproportionate fat loss, and ADSC‐ROS‐collagen cascade: Oxidative stress drives skin aging independently of fat loss. (B) Dual signaling: GLP‐1R and IGF‐1R pathways explain tissue selectivity.

While inducing facial lipodystrophy, GLP‐1RAs have been shown to accelerate wound healing, reduce fibrosis, and improve inflammatory skin conditions such as psoriasis and hidradenitis suppurativa [4]. This duality raises a fundamental question: does facial aging reflect cell‐type‐specific and depot‐specific differences in GLP‐1R expression and signaling pathways? According to Kruglikov, the local effects of glucagon‐like peptide‐1 receptor agonists are realized through two parallel signaling routes: a canonical pathway dependent on GLP‐1 receptor internalization, and a non‐canonical pathway mediated by the insulin‐like growth factor 1 receptor [5] (Figure 1B).

The facial changes described above have not emerged as safety signals in large‐scale randomized controlled trials of GLP‐1RAs. The predisposing clinical characteristics, including baseline facial fat volume, age, sex, rate of weight loss, and total weight loss percentage, remain uncertain. Additionally, it is essential to acknowledge that GLP‐1RAs provide substantial cardiovascular and renal benefits, including reductions in major adverse cardiovascular events, cardiovascular mortality, hospitalization for heart failure, and progression of chronic kidney disease. These benefits should not be overshadowed by cosmetic considerations in appropriately selected patients.

The current understanding of “Ozempic face” is therefore incomplete. The disproportionate fat loss, the oxidative stress‐collagen degradation cascade, and the paradoxical tissue selectivity all suggest that GLP‐1RAs compromise dermal health beyond what can be corrected with volume restoration alone. Until further evidence emerges, dermatologists should counsel patients that facial aging may involve more than simple fat loss, and that preventive strategies warrant investigation rather than relying solely on reactive fillers.

## Funding

The authors have nothing to report.

## Disclosure

Statement on Use of Artificial Intelligence in Manuscript Composition: No artificial intelligence or AI‐assisted technologies were used in the preparation of this manuscript.

## Ethics Statement

The manuscript does not report original human or animal research requiring IRB approval.

## Consent

The authors have nothing to report.

## Conflicts of Interest

The authors declare no conflicts of interest.

## Data Availability

Data sharing not applicable to this article as no datasets were generated or analysed during the current study.
